# Seroprevalence of hepatitis B and associated factors among inmates: a cross sectional study in the Douala New Bell Prison, Cameroon

**DOI:** 10.11604/pamj.2021.38.355.20386

**Published:** 2021-04-13

**Authors:** Mathurin Pierre Kowo, Firmin Ankouane Andoulo, Daniel Tchamdeu Sizimboue, Antonin Wilson Ndjitoyap Ndam, Larry Tangie Ngek, Charles Kouanfack, Hubert Leundji, Rocard Djanteng, Bruno Ela Ondo, Judith Ndongo Torimiro, Elie-Claude Ndjitoyap Ndam, Oudou Njoya

**Affiliations:** 1 Faculty of Medicine and Biomedical Sciences, University of Yaoundé 1, Yaoundé, Cameroon,; 2 Université des Montagnes, Bangangté, Cameroon,; 3 Faculty of Medicine and Pharmaceutical Sciences, University of Dschang, Dschang, Cameroon,; 4 Centre Médical de L'Estuaire Douala, Douala, Cameroon,; 5 Medical Center, New Bell Prison, Douala, Cameroon

**Keywords:** Prevalence, risks factors, hepatitis B, prison, Cameroon

## Abstract

**Introduction:**

in Cameroon, data on viral hepatitis B infection in prison environments is limited. We determined the prevalence of hepatitis B infection (HBV) and correlates among prisoners incarcerated at the Douala New Bell Central Prison in Cameroon.

**Methods:**

this was a cross-sectional study carried out in July 2018 and included 940 randomly selected prisoners. Data were collected using pre-tested questionnaire while blood screening for HBV surface antigen (HBs Ag) used rapid test, with confirmation via Elisa test. Sociodemographic characteristics and risk factors were compared among the three age groups with respect to the prison's partitioning. Factors associated with positive HBs Ag were identified using logistic regression adjusted to age and gender. Confounders were then excluded by logistic multivariate analysis. All p values less than 0.05 were considered statistically significant.

**Results:**

of the 940 prisoners selected, 94% were male. The mean age of the study population was 33.81 ± 10.35 years. The median duration of incarceration and median number of incarcerations were 12 months (IQR: 5-36) and 1 (IQR: 1-2) respectively. HBV prevalence was 12.9% (95% CI: 10.7-15%). The use of non-injectable illicit drugs (OR: 3.5; 95% CI: 1.9-6.2; P<0.001), sharing of needle or razors (aOR: 24.1; 95% CI: 12.9-45.0; P<0.001), sharing of tooth brushes(aOR: 2.7; 95% CI: 0.9-7.4) (P=0.053), having tattoos or piercings (aOR: 1.9; 95% CI: 1.1-3.1; P=0.01) were significantly associated with HBs Ag seropositivity.

**Conclusion:**

prisoners in this setting had a high prevalence of HBV and related risk factors. These findings highlight an urgent need to implement control strategies and programs that reach people in detention centers in Cameroon.

## Introduction

Hepatitis B virus (HBV) infection is a major global public health problem, with significant mortality and morbidity especially in sub-Saharan African and East Asian countries [1]. Globally, in 2015, 257 million people were living with chronic HBV infection according to the World Health Organization (WHO) estimates [1]. The African and Western Pacific regions accounted for 68% of those infected. The number of HBV related deaths is about 650,000 every year mainly due to liver cirrhosis and/or hepatocellular carcinoma (HCC) [2].

A recently publish systematic review and meta-analysis on seroprevalence of hepatitis B virus infection in Cameroon has estimated the overall pooled prevalence of HBs Ag at 11.2%, making Cameroon one of the most affected countries in the world [3]. Prisoners are a high-risk population for infectious diseases with a high prevalence for viral hepatitis and HIV compared to the general population [4]. The overall prevalence of HBs Ag ranges from 0.3% to 25.5% has been reported by several studies in Europe [5], North America [6], Latin America [7] and Africa [8,9] among adult inmates. This high risk is linked to the prison setting with poor hygiene, promiscuity, malnutrition, tattooing and other forms of skin piercing, physical and moral violence, intravenous drug use, males having sex with males and lack of knowledge about HBV transmission modes [10,11].

In addition, prisoners suffered from limited access to appropriate health care services, lack of early detection and treatment of some chronic diseases and could serve as reservoirs for HBV infection. Despite the high prevalence of HBV infection in Cameroon, published data on the burden in prison settings are lacking. The aim of this study was to determine the prevalence of hepatitis B surface antigen and correlates among inmates of the Douala New Bell central prison in Cameroon.

## Methods

**Study design:** this was a cross sectional study conducted from November 2017 to October 2018 at the Douala New Bell Central Prison. It included 940 inmates randomly selected from a total of 3356 inmates, using systematic random sampling. This was based on the alphabetical list of prisoners present at the start of the study, applying a sampling interval of 4. Data collected included age, gender, profession before detention, level of education, marital status, religion, number and duration of incarcerations. Data on risk factors for HBV transmission were also collected on a pretested questionnaire. Rapid detection of HBsAg was done using an immunochromatography assay (HEPATM (HBs Ag) strip test) (RECKON DIAGNOSTICS P.LTD) (3/7, BIDC, Gorwa Vadogara 390 016 (INDIA)) in accordance with the manufacturer´s instruction. This rapid test is used for qualitative detection of HBs Ag in serum and plasma. Inmates tested positive on the rapid test had a confirmatory Elisa test.

**Study setting:** the New Bell Central Prison is located in the New-Bell suburb of Douala (Cameroon). It was created in 1930 and is the main prison for the Littoral Region of Cameroon. It constitutes with the Kondengui Central Prison Yaoundé the 2 main central prisons in Cameroon. It is a mixed prison with separate male, female and children quarters. It has a capacity of 960 places for over 3500 prisoners with 27 cells containing between 30-200 prisoners per cell giving an average space of 0.2m^2^ per inmate for a minimum international standard of 4m^2^. The New Bell Central Prison is divided into 10 quarters for 4 different groups of persons: minors (males below 18), the elderly (males above 55), females of all age groups and males between 18 and 55. Males between 18 and 55 constitute 86.7% of all inmates. The prison offers health services for inmates and has one medical doctor, two senior nurses, five assistant nurses and two laboratory technicians. All new prisoners have complete physical examination by the site physician with systematic human immunodeficiency virus (HIV) screening but no screening for viral hepatitis.

**Data analysis:** data were analyzed using the Statistical Package for Social Sciences (SPSS) version 21.0 (Institute, Cary, NC, USA). The results were expressed in numbers and percentages for qualitative variables, mean ± SD for normally distributed variables, median with interquartile range (IQR) for non-normally distributed variables. Sociodemographic and risk factors were described and compared across three age groups with respect to the prison´s partitioning (children below 18, adults between 18 and 55 and the elderly above 55) using the Chi-square test. Factors associated to positive HBs Antigen were identified using logistic regression adjusted to age and gender. The odds ratio (95% CI) were calculated to assess the strength of association. Confounders were then excluded by logistic multivariate analysis including age, gender and all significant variables. P values less than 0.05 were considered statistically significant.

**Ethical considerations:** ethical clearance was obtained from the institutional ethical review board of the Université des Montagnes-Bangangté, Cameroon, while administrative authorization was obtained from the Regional Delegate for Penitentiary Administration for the Littoral Region and the Superintendent in charge of the New Bell Central Prison. Prison inmates who tested positive for HBs antigen were referred to the medical doctor in charge of the prison health service for proper management. All included inmates signed a written consent form.

## Results

Of the 940 prisoners included, 94% (884) were males and 6% (56) were females. The mean age was 33.81 years ± 10.35 years with extremes of 14 and 74 years. The most represented age group was 30-39 years. The median duration of incarceration and median number of incarcerations were 12 months (IQR: 5-36) and 1 (IQR: 1-2) respectively (Table 1). The prevalence of HBs Ag was 12.9% (95% CI: 10.7-15%) and did not significantly differ among age groups (p=0.86) (Table 2). Among 940 prisoners, 60% (566) had never heard of viral hepatitis before this study. The vaccination coverage rate for HBV was 4.3% (n=40) (Table 3).

**Table 1:** socio-demographic and carceral characteristics

Variables	Overall (n=940)	<18 yrs (n=37)	18 - 55 yrs (n=858)	> 55 yrs (n=45)	P value
**Gender**					
Male	884 (94)	36 (97.3)	805 (93.8)	43 (95.6)	0.620
Female	56 (6)	1 (2.7)	53 (6.2)	2 (4.4)	
**Level of education**					0.011
No formal education	629 (66.9)	17 (45.9)	578 (67.4)	34 (75.6)	
Secondary and above	311 (33.1)	20 (54.1)	280 (32.6)	11 (24.4)	
**Marital status**					<0.001
Single	560 (59.6)	37 (100)	516 (60.1)	7 (15.6)	
Married	368 (39.1)	0 (0)	334 (38.9)	34 (75.6)	
Widow	12 (1.3)	0 (0)	8 (0.9)	4 (8.9)	
**Religion**					0.790
Christian	734 (78.1)	28 (75.7)	668 (77.9)	38 (84.4)	
Muslim	200 (21.3)	9 (24.3)	184 (21.4)	7 (15.6)	
Others	6 (0.6)	0 (0)	6 (0.7)	0 (0)	
**Former profession**					<0.001
Private sector	400 (42.6)	5 (13.5)	372 (43.4)	23 (51.1)	
Civil servant	114 (12.1)	0 (0)	107 (12.5)	7 (15.6)	
Trader	193 (20.5)	4 (0.8)	182 (21.2)	7 (15.6)	
Student	99 (10.5)	24 (64.9)	75 (8.7)	0 (0)	
Farmer	25 (2.7)	0 (0)	22 (2.6)	3 (6.7)	
Unemployed	109 (11.6)	4 (10.8)	100 (11.7)	5 (11.1)	
**Duration of incarceration (months)**					0.001
<12	413 (43.9)	23 (62.2)	380 (44.3)	10 (22.2)	
≥12	527 (56.1)	14 (37.8)	478 (55.7)	35 (77.8)	
Median (IQR)	12 (5 - 36)	5 (3 - 18.5)	12 (5 - 36)	19 (12 - 63)	0.002
**Number of incarcerations**					0.003
1	690 (73.4)	36 (97.3)	619 (72.1)	35 (77.8)	
≥ 2	250 (26.6)	1 (2.7)	239 (27.9)	10 (22.2)	
Median (IQR)	1 (1 - 2)	1 (1 - 1)	1 (1 - 2)	1 (1 - 1)	0.003

**Table 2:** prevalence of HBs Antigen positivity

Variables	Overall (n=940)	<18 yrs (n=37)	18 - 55 yrs (n=858)	>55 yrs (n=45)	P value
HBsAg					0.865
Positive	121 (12.9)	4 (10.8)	112 (13.1)	5 (11.1)	
Negative	819 (87.1)	33 (89.2)	746 (86.9)	40 (88.9)	

Overall prevalence: 12.9% (95% CI: 10.7 - 15%)

**Table 3:** risk factors for HBV

Variables	Overall (n=940)	< 18 yrs (n=37)	18 - 55 yrs (n=858)	>55 yrs (n=45)	P value
Poor knowledge on HBV transmission	625 (66.5)	33 (89.2)	567 (66.1)	25 (55.6)	0.004
Vaccination against HBV	40 (4.3)	0 (0)	38 (4.4)	2 (4.4)	0.425
Injected illicit drugs used	26 (2.8)	1 (2.7)	25 (2.9)	0 (0)	0.509
Non injected illicit drugs used	431 (45.9)	25 (67.6)	388 (45.2)	18 (40)	0.020
Men having sex with men (n=884)	32 (3.6)	1 (2.8)	29 (3.6)	2 (4.7)	0.903
Sharing of needles or razors	271 (28.8)	11 (29.7)	244 (28.4)	16 (35.6)	0.585
Sharing of teeth brushes	42 (4.5)	4 (10.8)	38 (4.4)	0 (0)	0.061
History of tattooing or piercing	360 (38.3)	18 (48.6)	327 (37.1)	15 (33.3)	0.340
History of blood transfusion	115 (12.2)	9 (24.3)	103 (12)	3 (6.7)	0.041
History of scarification	391 (41.6)	23 (62.2)	348 (40.6)	20 (44.4)	0.031

Out of the 940 inmates, 66.9% (629) had no formal education and this was not associated with HBs Ag positivity (aOR: 0.9; 95% CI: 0.5-1.7; P=0.859). No association was found between HBs Ag positivity and gender (aOR: 1.7; 95% CI: 0.7-4.5; P=0.246) although they were more HBs Ag positivity in female (Table 4). The use of non-injectable illicit drugs (OR: 3.5; 95% CI: 1.9-6.2; P<0.001), sharing of needle or razors (aOR: 24.1; 95% CI: 12.9-45.0; P<0.001), sharing of tooth brushes (aOR: 2.7; 95% CI: 0.9-7.4) (P=0.053), having tattoos or piercings (aOR: 1.9; 95% CI: 1.1-3.1; P=0.01) were significantly associated with HBs antigen seropositivity (Table 5).

**Table 4:** odd ratio for HBs Ag positivity according to socio-demographic and carceral characteristics among New Bell Prison inmates in Douala

Variables	Number tested (n=940)	HbsAg positive	HbsAg negative	OR (95% CI) £	P value£	OR (95% CI)*	P value*
**Gender**							
Male	884	112 (12.7)	772 (87.3)	1	1	1	1
Female	56	9 (16.1)	47 (83.9)	1.3 (0.6 - 2.7)	0.459	1.7 (0.7 - 4.5)	0.246
**Age range**							
< 18 yrs	37	4 (10.8)	33 (89.2)	1	1	1	1
18 - 55	858	112 (13.1)	746 (86.9)	1.2 (0.4 - 3.5)	0.706	2.7 (0.7 - 9.9)	0.139
> 55	45	5 (11.1)	40 (88.9)	1.03 (0.3-4.1)	0.971	1.2 (0.2 - 6.5)	0.830
**Level of education**							
No formal education	629	96 (15.3)	533 (84.7)	1	1	1	1
Secondary and above	311	25 (8)	286 (92)	0.5 (0.3 - 0.8)	0.002	0.9 (0.5 - 1.7)	0.859
**Marital status**							
Single	560	72 (12.9)	488 (87.1)	1	1		
Married	368	47 (12.8)	321 (87.2)	1.1 (0.7 - 1.6)	0.797		
Widow	12	2 (16.7)	10 (8.3)	1.5 (0.3 - 7.03)	0.638		
**Former profession**							
Private sector	400	55 (13.8)	345 (86.3)	1	1		
Civil servant	114	11 (9.6)	103 (90.4)	0.7 (0.3 - 1.3)	0.656		
Trader	193	26 (13.5)	167 (86.5)	0.9 (0.6 - 1.6)	0.872		
Student	99	9 (9.1)	90 (90.9)	0.6 (0.3 - 1.2)	0.161		
Farmer	25	1 (4)	24 (96)	0.3 (0.04 - 1.9)	0.193		
Unemployed	109	19 (17.4)	90 (82.6)	1.3 (0.7 - 2.3)	0.426		
**Duration of incarceration (months)**							
< 12	413	58 (14)	355 (86)	1	1		
≥ 12	527	63 (12)	464 (88)	0.8 (0.6 - 1.3)	0.405		
**Number of incarcerations**							
1	690	85 (12.3)	605 (87.7)	1	1		
≥ 2	250	36 (14.4)	214 (85.6)	1.2 (0.8 - 1.9)	0.364		

£Adjusted for age and gender ; *Adjusted for age, gender and other significant associated factors

**Table 5:** odd ratio for HBs Ag positivity according to risk factors among New Bell prison inmates in Douala

Variables	Number tested (n=940)	HbsAg positive	HbsAg negative	OR (95% CI) £	P value£	OR (95% CI)*	P value*
**Poor knowledge on HBV transmission**							
Yes	625	83 (13.3)	542 (86.7)	1.1 (0.7 - 1.7)	0.567		
No	315	38 (12.1)	277 (87.9)	1	1		
**Vaccination against HBV**							
Yes	40	4 (10)	36 (90)	0.7 (0.3 - 2.1)	0.581		
No	900	117 (13)	783 (87)	1	1		
**Injectable illicit drugs used**							
Yes	26	7 (26.9)	19 (73.1)	2.5 (1.03 - 6.2)	0.041	1.3 (0.4 - 4.03)	0.595
No	914	114 (12.5)	800 (87.5)	1	1	1	1
**Non injectable illicit drugs used**							
Yes	431	100 (23.2)	331 (76.8)	7.6 (4.6 - 12.6)	< 0.001	3.5 (1.9 - 6.2)	< 0.001
No	509	21 (4.1)	488 (95.9)	1	1	1	1
**Men having sex with men**							
Yes	32	9 (28.1)	23 (71.9)	2.9 (1.3 - 6.4)	0.009	1.2 (0.4 - 3.4)	0.694
No	852	103 (12.1)	749 (87.9)	1	1	1	1
**Sharing of needles or razors**							
Yes	271	108 (39.9)	163 (60.1)	33.4 (18.3 - 60.8)	< 0.001	24.1 (12.9 - 45.01)	< 0.001
No	669	13 (1.9)	656 (98.1)	1	1	1	1
**Sharing of teeth brushes**							
Yes	42	12 (28.6)	30 (71.4)	2.9 (1.4 - 5.8)	0.003	2.7 (0.9 - 7.4)	0.053
No	898	109 (12.1)	789 (87.9)	1	1	1	1
**History of tattooing or piercing**							
Yes	360	81 (22.5)	279 (77.5)	3.9 (2.6 - 6)	< 0.001	1.9 (1.1 - 3.1)	0.015
No	580	40 (6.9)	540 (93.1)	1	1	1	1
**History of blood transfusion**							
Yes	115	16 (13.9)	99 (86.1)	1.1 (0.6 - 1.9)	0.697		
No	825	105 (12.7)	720 (87.3)	1	1		
**History of scarification**							
Yes	391	73 (18.7)	318 (81.3)	2.4 (1.6 - 3.6)	< 0.001	1.3 (0.8 - 2.1)	0.292
No	549	48 (8.7)	501 (91.3)	1	1	1	1

£Adjusted for age and gender; *Adjusted for age, gender and other significant associated factors

## Discussion

Overcrowding in prisons remains a major problem in both developed and developing countries and is a key factor for a myriad of other problems which ultimately turn these custodial settings into fertile breeding grounds for infectious diseases such as viral hepatitis, HIV, syphilis, gonorrhea and tuberculosis [4,8]. Knowledge of the prevalence of HBV infection and associated factors in prisoners is therefore an important step for the planning and implementation of preventive measures in this population. This study aimed to determine the prevalence of HBV infection and correlates among prison inmates in Cameroon and addressed issues for preventive measures in this setting. The prevalence of chronic HBV infection in our study population was 12.9%. This prevalence is higher than that of the general population in Cameroon recently estimated at 7.1% (95% CI: 2.6-13.5%) [3]. Our result confirms the high endemicity of HBV infection in Cameroon involving both the general population and special population such as individuals who are detained.

Similar high prevalences of HBV infection in prisoners, higher than that of general population from which they arise have been reported among inmates in many other parts of the World [4,8,9,12-14]. In high endemicity area like sub-Saharan Africa and East Asian Countries, high prevalence of the HBV infection has mainly been attributed to HBV transmission occurring predominantly early in the life either through mother to child transmission and horizontal transmission, low HBV vaccine coverage, unsafe medical and traditional procedures [15,16]. The high prevalence of HBV in prisoners in Cameroon found in this study suggests the presence of some additional risk factors that could explained the spread of this infection in prisons.

In the present study, the use of non-injectable illicit drugs but not injectable illicit drugs was associated to HBV infection. This finding is however, paradoxical as several authors have reported an association between the use of injectable illicit drugs and HBV infection [17,18]. This finding could be attributed to the following reasons: an underestimation of the use of injectable illicit drugs by inmates because of oral interview as a means of data collection, difficulties in acquiring injectable materials in the prison setting and the absence of complete physical examination to assess for injection site. Sharing of needles and razors, past medical history of tattooing/piercings were also significantly associated to HBV infection. HBV is a highly infectious agent which can remain stable on environmental surfaces for 7 days [19] and practices such as sharing of needles and razors, tattoos or piercing and scarifications found respectively in 28.8%, 38.3%; 41.6% of our study population may have played a role in the diffusion of the virus in this population. However, whether these practices are more frequent in the Cameroonian prisoners than the general population is still unknown.

We also demonstrated that the anti-HBV vaccination coverage rate was low at 4.3%. The low anti-HBV vaccination rate in this high-risk population could be explained by the absence of policy of screening and vaccination of these high-risk individuals in Cameroon. In effect, safe and effective vaccines against hepatitis B have been available since 1982. This vaccine has been shown to be effective in 95% of cases in preventing individuals from developing chronic infections and in reducing the incidence of related complications [20]. Since 1990, the WHO recommends introduction of HBV vaccine into the expanded program of immunization in member states [21]. Unfortunately, in Cameroon, this vaccine was only introduced in the year 2005 as an integral part of the routine expanded program of immunization at 6, 10 and 14 weeks after delivery. If this measure represents an important step to control HBV infection in Cameroon, it fails to protect the adult populations especially those at risk of HBV infection as there is no HBV catch-up vaccination policy for this population.

The low vaccination coverage rate found in detainees in Cameroon is consistent with that reported in another risk population by Desmond Aroke *et al*. who reported vaccination coverage rate of only 16.8% among Cameroonian medical students [22]. These results emphasize the urgent need to implement systematic screening and catch-up vaccination policies in risk populations for HBV infection in Cameroon. Low level of education was not associated with HBV transmission. The link between the level of education and the risk of transmission of HBV is still a matter of controversy. Some studies [23,24] have demonstrated a clear link between low education and HBV infection and others did not [5]. To the best of our knowledge, this study is the first addressing the issue of HBV infection among prison inmates in Cameroon. This study was also done in one of the major central prisons in Cameroon accommodating more than three thousand inmates. The high prevalence of HBV infection reported in this study and the associated factors highlight the necessity of implementing preventive measures dedicated to this population. However, due to the unicentric nature of this study these findings should be interpreted with caution. Further studies involving multiple prison centers is encouraged.

## Conclusion

Inmates in New Bell Central Prison accumulate risk factors for viral hepatitis thus contributing to the high prevalence in this setting. There is therefore an urgent need to implement a viable health policy to control HBV infection in this population.

### What is known about this topic

- Viral hepatitis is common in the prison setting;
- Multiple risk factors for HBV transmission exists in the prison setting;
- Publish data in Cameroon is rare.

### What this study adds

- It gives HBV prevalence and risk factors in the prison setting in Cameroon;
- Emphasizes the importance of a structured health policy for systematic screening for viral hepatitis and vaccination for negative inmates.
